# Is It Possible to Trap 4HNE, the Main Degradation Product of Omega-6? A Theoretical Study of Potential Scavengers

**DOI:** 10.1021/acsomega.6c02527

**Published:** 2026-04-30

**Authors:** Rodrigo Ramírez, Ana Martínez

**Affiliations:** Departamento de Materiales de Baja Dimensionalidad, Instituto de Investigaciones en Materiales, 7180 Universidad Nacional Autónoma de Mexico, Circuito Exterior S.N. Ciudad Universitaria, Ciudad de Mexico 03900, Mexico.

## Abstract

Omega-6 is a polyunsaturated fatty acid with potential therapeutic applications in humans. The main degradation product of Omega-6 is the α–β-unsaturated aldehyde 4-hydroxynonenal (4HNE). This compound induces DNA damage by forming covalent adducts with nitrogenous bases. To prevent this, there are strategies to trap this molecule with scavengers. The main scavengers are phloretin (Phr) and hesperetin dihydrochalcone (HDC). In this research, we investigate 4HNE forming covalent bonds with scavengers (Phr and HDC) and with nitrogenous bases (thymine, guanine, cytosine, and adenine). We first analyze the electron transfer properties, seeking a correlation between chemical parameters and the toxicity of 4HNE. We obtained the stabilization energies and electron transfer properties of the adducts. The question is whether the adducts with 4HNE can maintain similar electron transfer properties. The stabilization energies of 4HNE with the scavengers are similar to the stabilization energy with guanine (approximately 30–40 kcal/mol). The adducts are worse electron acceptors than 4HNE. This means that the adducts are worse oxidants than 4HNE, which is an advantage. The ability of 4HNE to oxidize nitrogenous bases decreases when the adducts are formed. The scavenger capacity of Phr and HDC reduces the toxicity of 4HNE for two reasons: they trap 4HNE, preventing it from reacting with biomolecules, and the adducts formed are not as effective oxidants as 4HNE, resulting in less damage to biomolecules.

## Graphical abstract

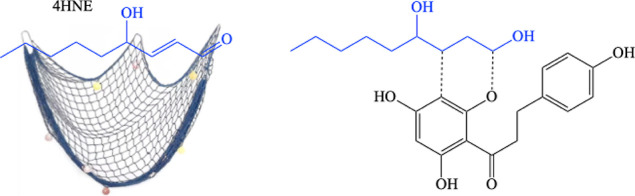

## Introduction

Omega-6 is a polyunsaturated fatty acid that has become very popular due to its potential therapeutic applications in humans.^1–35^ It is a chain of carbon atoms with one carboxyl group at one end of the chain and a methyl group at the other, with two or more double bonds between the carbons within the fatty acid chain.^23^ The number in the name of Omega indicates the position of the carbon–carbon double bond counted from the methyl end of the chain. In the case of Omega-6, it is located at the sixth carbon atom from the methyl end of the chain. Fatty acids are crucial since they are the main building blocks of cell membranes (lipid bilayer).^22^ Mammals cannot produce Omega-6 endogenously; therefore, they must obtain it mainly from food and, occasionally, as a nutritional supplement. Omega-6 appears to be essential for preventing some serious diseases.

Like most supplements and medications, Omega-6 is degraded during metabolism.^36–49^ One of the major degradation products of Omega-6 is the α–β unsaturated aldehyde 4-hydroxynonenal (4HNE) (Figure 1a). The presence of 4HNE in the human body has been linked to chronic diseases, including cancer, neurodegenerative processes, diabetes, cardiovascular disease, and Alzheimer’s disorder.^40–47^ 4HNE is a major, highly reactive lipid peroxidation product that damages proteins, nucleic acids, and lipids. It is one of the most abundant and widely studied cytotoxic products of lipid peroxidation.^48–54^ 4HNE has been reported to induce DNA damage by forming covalent adducts with nitrogenous bases.^53,54^ This produces structural disturbances in the DNA molecule that can cause strand breakage or chemical changes in the base.

**Figure 1:**
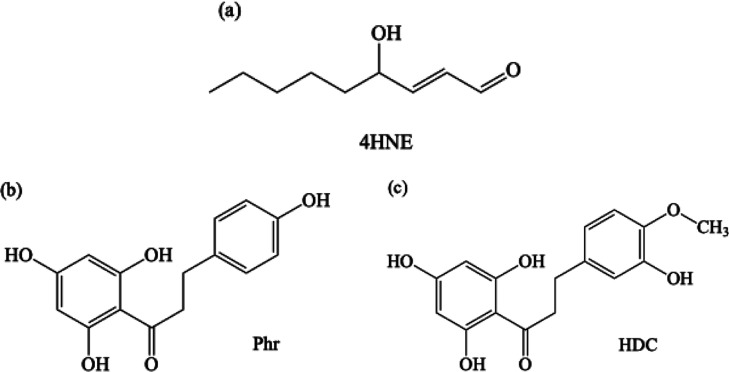
Schematic representation of (a) α–β unsaturated aldehyde 4-hydroxynonenal (4HNE), (b) phloretin (Phr), and (c) hesperetin dihydrochalcone (HDC).

Omega-6 is essential for human health but produces 4HNE, which is toxic. To prevent toxicity, one possibility is to avoid Omega-6 intake, but this is not the best solution, as we would be wasting the health benefits that this molecule provides. The other possibility is to address the problem of 4HNE. Due to the potential damage this molecule could cause, it is important to find strategies to control its presence in the organism. One of the most popular strategies is to trap this molecule with different agents such as phloretin (Phr) and hesperetin dihydrochalcone (HDC).^50–53^ Phr and HDC are quite similar molecules (see Figure 1b,c) and some authors suggest that both could trap 4HNE.^50,51^

Phr and HDC trap 4HNE by forming covalent bonds. 4HNE also forms covalent bonds with DNA bases. Apparently, there could be competition between the scavengers and the nitrogenous bases of DNA. The question here is which bond is stronger. Despite all the reported results on the reactivity of 4HNE, there are no studies that consider the strength of the covalent bonds that 4HNE might form with the scavengers and with the nitrogenous bases of DNA. For this reason, the main objective of this investigation is to analyze the covalent bonds that 4HNE forms with the scavengers (Phr and HDC) and with nitrogenous bases (thymine, guanine, cytosine, and adenine). To do this, we first analyzed the electron transfer properties, seeking a correlation between the chemical parameters and the toxicity of 4HNE. After this analysis, we obtained the stabilization energies and also the electron transfer properties of the adducts formed. The question is whether the adducts formed with 4HNE could maintain similar electron transfer properties. With these results, it is possible to obtain more information about the chemical reactivity of 4HNE, which is the main degradation product of Omega-6.

## Methods

### Computational Details

Initial geometries were obtained from PubChem databases.^55^ Gaussian16 was used for all electronic calculations.^56^ Geometry optimizations of initial geometries were obtained at the M05-2*X*/6-311 + g­(2d,p) level of theory without symmetry constraints.^57–60^ Local minima were confirmed by the absence of imaginary frequencies. SMD was used as a solvation model using water and benzene to mimic a polar and no polar environment, respectively.^61^ SMD is a solvation model that can be applied to any charged or uncharged solute in any solvent or liquid medium. M05-2X is a global hybrid exchange–correlation GGA approach to Density Functional Theory, designed for thermochemistry, kinetics, and covalent interactions.

To analyze the electron transfer properties, it is possible to use electron affinity (A) and ionization potential (I). I and A are the vertical ionization energy and vertical electron affinity, respectively, obtained as follows:

1 $$Y{\to Y}^{+1}+{1e}^{-}I=E(Y^{+1})-E\left(Y\right)$$

2 $$Y^{-1}\to Y+{1e}^{-}A=E\left(Y\right)-E(Y^{-1})$$

Optimized structures of neutral systems (represented by Y) were used to calculate the single point energies of the cation (Y^+^) and the anion (Y^–^).

Low values of I indicate good electron donor molecules. High values of A are for good electron acceptor molecules. With these parameters, it is possible to determine as indicated in Figure 2 the Full Electron Donor–Acceptor Map (FEDAM).^62,63^ Systems located down to the left are considered good electron donors and therefore good reductants, while those situated up to the right are good electron acceptors or good oxidants. These quantities allow us to describe the redox properties of the systems and have been successfully used in various chemical systems.^62–74^

**Figure 2:**
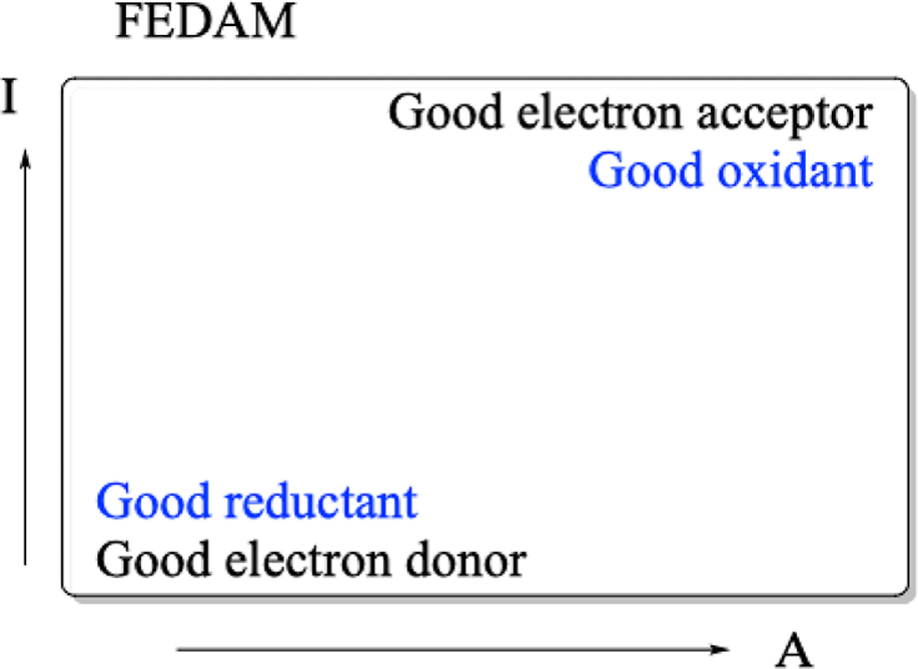
Full Electron Donor–Acceptor Map (FEDAM).

To investigate the stability of the adducts, we optimized the geometries and calculated the stabilization energy (Δ*E*) as follows:

3 ΔE(4HNE−X)=[E(4HNE)−E(X)]−E(4HNE−X)

X is HDC, Phr, or nitrogenous bases. Positive values imply that adducts are more stable than the separated compounds.

## Results and Discussion

Figure 3 reports the optimized structures in water and benzene of 4HNE, Phr, and HDC. Due to the single C–C bonds, all stable structures are twisted. Optimized geometries are similar in water and benzene. To analyze the electron transfer properties, in Figure 4, we report the FEDAM of these three molecules in water and benzene to mimic polar and nonpolar environments. DNA nitrogenous bases [thymine (T), guanine (G), cytosine (C), and adenine (A)] are also reported. The formed adducts that we will analyze later are also included.

**Figure 3:**
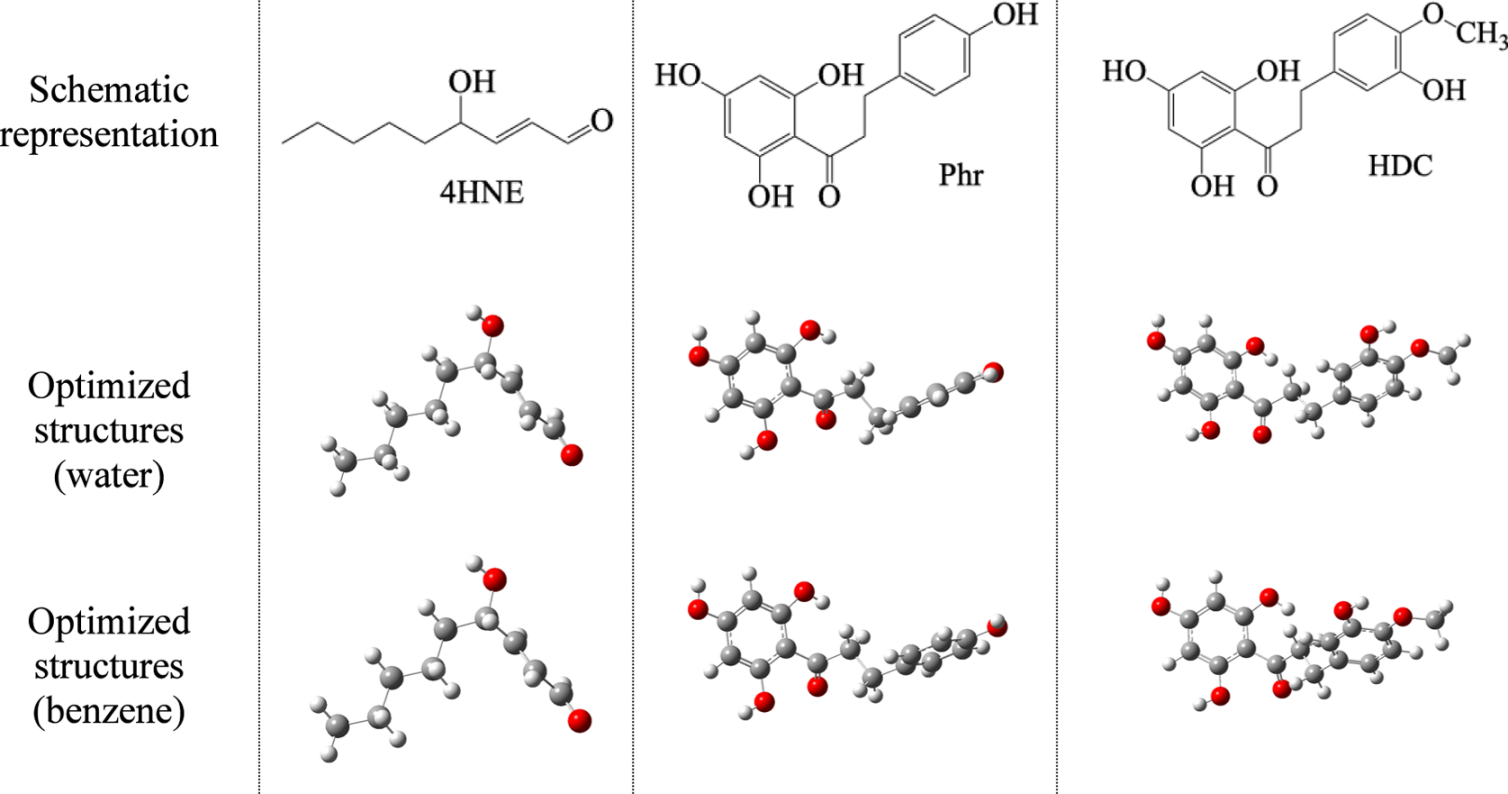
Schematic representation and optimized structures in water and benzene of α–β unsaturated aldehyde 4-hydroxynonenal (4HNE), phloretin (Phr), and hesperetin dihydrochalcone (HDC). Gray spheres are C atoms; red spheres are O atoms.

**Figure 4:**
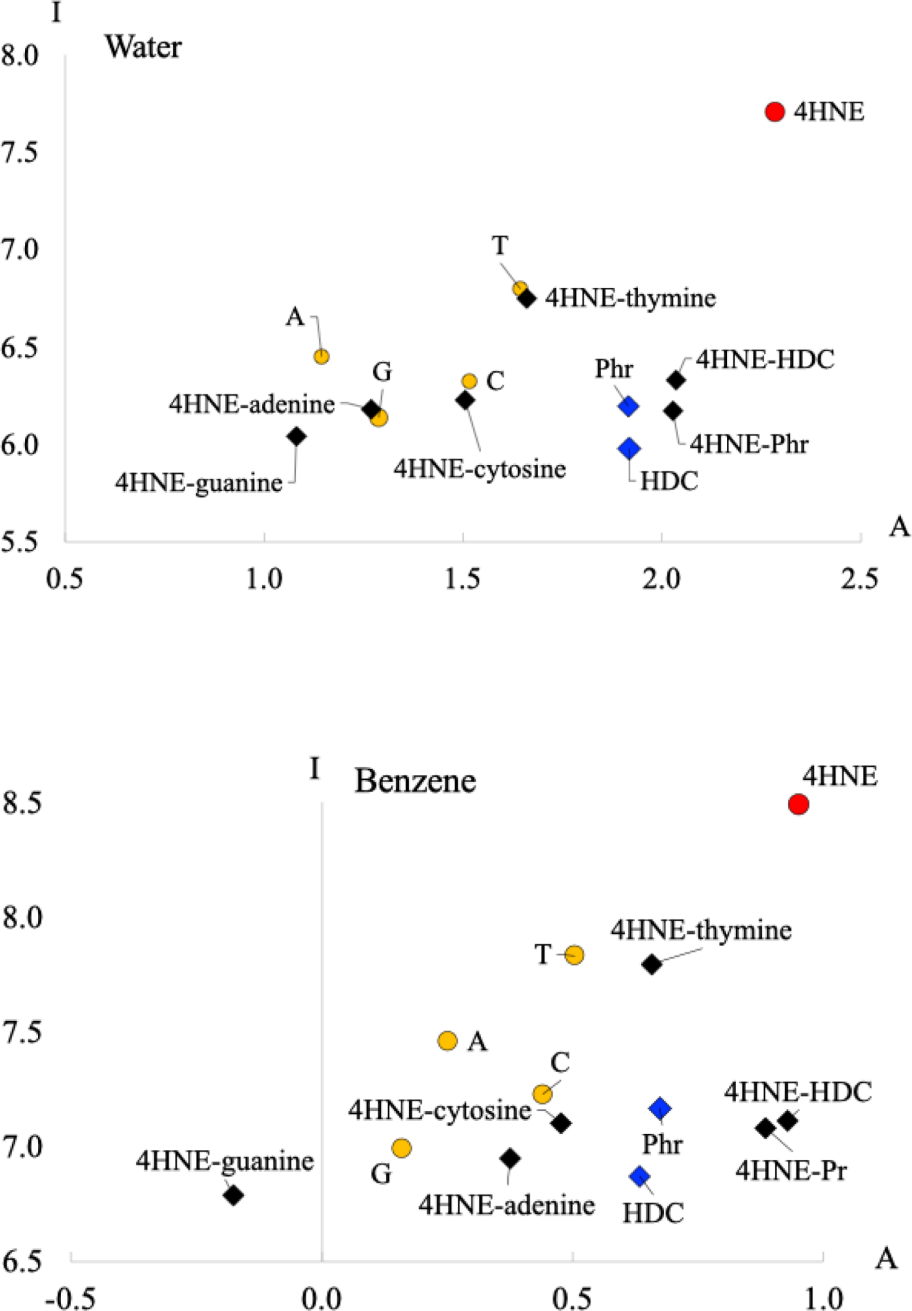
FEDAM (in water and benzene) of α–β unsaturated aldehyde 4-hydroxynonenal (4HNE), phloretin (Phr), hesperetin dihydrochalcone (HDC), thymine (T), guanine (G), adenine (A), and cytosine (C). The formed adducts that we will analyze later are also included. Values in eV.

According to Figure 4, 4HNE is the best electron acceptor in water and benzene. It can remove electrons from nitrogenous bases and also from HDC and Phr. Therefore, 4HNE is a good oxidant agent, as it will accept electrons from nitrogenous bases, and nitrogenous bases will be oxidized. This could be related to covalent modifications and could also be linked to chronic diseases. The main degradation product of Omega-6 could be toxic due to its capacity to oxidize biomolecules.

Comparing the scavengers in water, HDC is the best electron donor, and it is a good electron acceptor. Phr is a good electron acceptor and also a good electron donor. Both scavengers could donate electrons to 4HNE. The values of I for nitrogenous bases are close to those of HDC and Phr, with the exception of thymine, which is the worst electron donor among nitrogenous bases. Nitrogenous bases are worse electron acceptors than HDC and Phr. In benzene, HDC and Phr have similar electron donor–acceptor properties, and both are better electron acceptors than nitrogenous bases. This can be interpreted as Phr and HDC having a lower capacity to oxidize nitrogenous bases than 4HNE, since they are worse electron acceptors. Apparently, these two scavengers cannot transfer electrons to nitrogenous bases but they could transfer electrons to 4HNE.

A correlation between the electron acceptor properties and the potential toxicity has been reported.^70,71^ Molecules that are good electron acceptors can be toxic because they extract electrons from other molecules that lose them. The loss of electrons is the oxidation process, and therefore, good electron acceptors oxidize other molecules. Accordingly, in this research, the key finding regarding electron transfer properties is that 4HNE exhibits potential toxicity related to its ability to oxidize biomolecules, while the scavengers (HDC and Phr) are less able to oxidize them.

To trap 4HNE, scavengers must form stable covalent bonds with this degradation product. To investigate the scavenger capacity, we studied the adducts formed with 4HNE bonded to Phr and HDC. We selected three different initial geometries (Figure 5). These three positions are obtained from ref 50. Figures 5 and 6 report the optimized structures in water and benzene. Stabilization energies (eq 3) are also included. Δ*E* values are positive, which means that all products of the reactions are more stable than the separated reactants. Due to these results, HDC and Phr could trap 4HNE. The stabilization energies are larger than 25 kcal/mol for most stable compounds. The most stable adducts are 4HNE-HDC-P2 and 4HNE-Phr-P2, and the less stable ones are 4HNE-HDC-P1 and 4HNE-Phr-P1. This is a logical finding since these last structures have only one C–C bond between 4HNE and the scavenger, while the other two structures present also C–O bonds. The stabilization energy is related to the bond distances. All C–C bond distances between 4HNE and HDC or Phr are equal to 1.5 Å. Compounds in the P2 and P3 conformations have C–O bonds of 1.4 Å, while those in the P1 conformation do not present C–O bonds. This difference explains the stabilization energies, which are higher for P2 and P3 conformations than for the P1 conformation. All compounds have one hydrogen bond formed between 4HNE and HDC or Phr. The differences in stabilization energies between the P2 and P3 conformations are related to the distance of this hydrogen bond (and, therefore, its strength). The hydrogen bond distance of the P2 conformation is 1.6 Å, while it is 2.4 Å for the P3 conformation. This information reveals the nature of the bonds and explains the differences in the stabilization energies.

**Figure 5:**
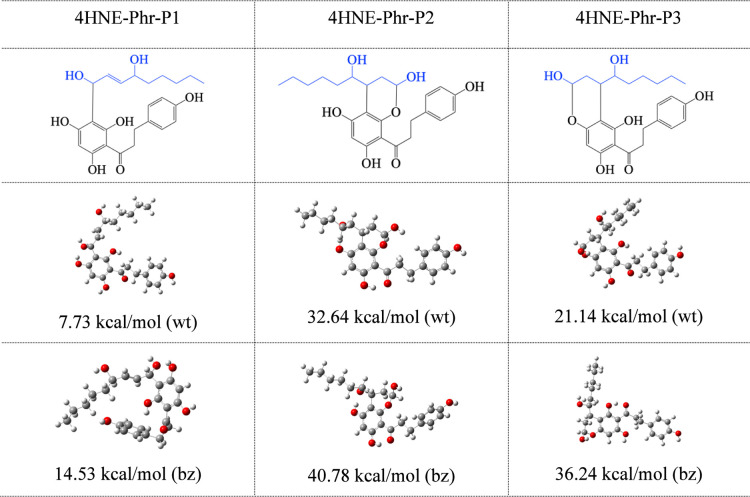
Schematic representation and optimized structures in water (wt) and benzene (bz) of adducts formed between 4HNE and Phr. Gray spheres are C atoms; red spheres are O atoms.

**Figure 6:**
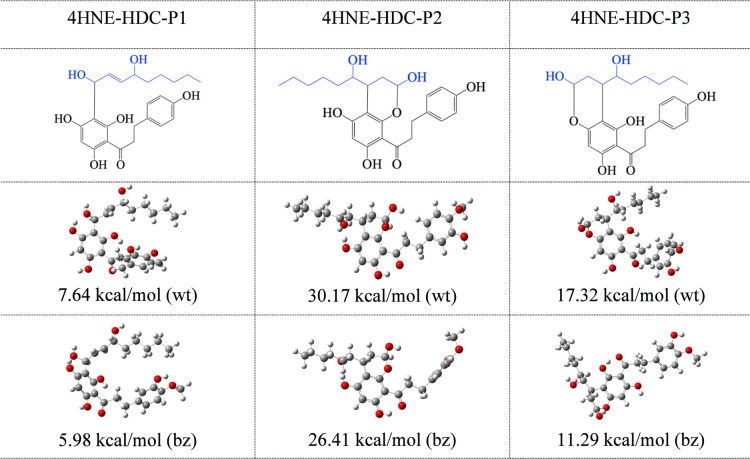
Schematic representation and optimized structures in water (wt) and benzene (bz) of adducts formed between 4HNE and HDC. Gray spheres are C atoms; red spheres are O atoms.

The formation of stable compounds with the scavengers indicates the 4HNE-scavenging ability of HDC and Phr, Phr being better than HDC since it forms stronger bonds with 4HNE. The most stable adducts, 4HNE-HDC-P2 and 4HNE-Phr-P2, are used in what follows to continue the analysis of the chemical reactivity.

To scavenge 4HNE, the stabilization energy with the scavengers must be greater than the stabilization energy of 4HNE with nitrogenous bases. Otherwise, it will be more stable to bond with the nitrogenous bases, and the scavengers will not be able to trap the 4HNE. To investigate the stability of the adducts with DNA bases, we optimized the geometries. Figure 7 reports the optimized structures and Δ*E* of four nitrogenous bases bonded to 4HNE.

**Figure 7:**
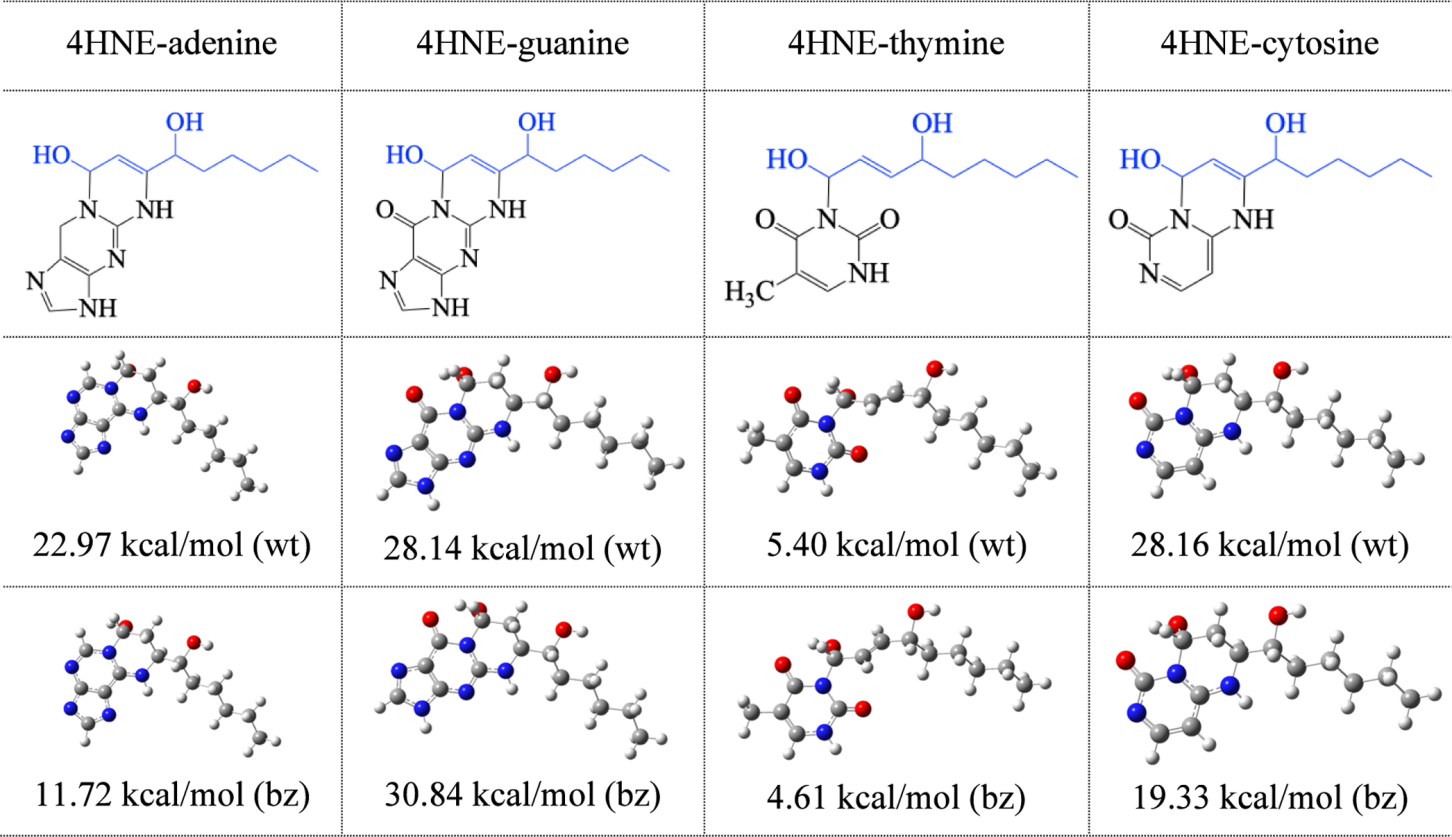
Schematic representation and optimized structures in water (wt) and benzene (bz) of 4HNE bounded to nitrogenous bases. Gray spheres are C atoms, red spheres are O atoms, and blue spheres are N atoms.

All nitrogenous bases form stable compounds with 4HNE. The results show that, with nitrogenous bases, 4HNE-thymine is the less stable product while 4HNE-guanine is the most stable in water and benzene. 4HNE-cytosine in water presents the same stabilization energy as 4HNE-guanine. The stability order with nitrogenous bases is 4HNE-guanine >4HNE-cytosine >4HNE-adenine >4HNE-thymine. This is in agreement with the reactivity of the aldehyde reported previously.^25^ When comparing the Δ*E* of Figures 5, 6, and 7, it is observed that the most stable systems with the highest stabilization energy are 4HNE-Phr-P2, 4HNE-HDC-P2, 4HNE-guanine, and 4HNE-cytosine in water. In benzene, they are 4HNE-Phr-P2, 4HNE-Phr-P3, and 4HNE-guanine. Since 4HNE and the scavengers present low solubility in water, the results of the stabilization energies in benzene become more important for the analysis. In benzene, Phr forms bonds 10 kcal/mol stronger than guanine, and 4HNE-HDC bonds are less stable than those with guanine (in benzene).

Based on these results, it is reasonable to assume that Phr will be a more effective scavenger than HDC, since it forms stronger bonds with 4HNE and also because 4HNE-HDC-P2 is less stable than 4HNE-guanine. Therefore, in the competition with the nitrogenous bases, HDC will be less effective. This is consistent with recent scientific evidence that suggests Phr stands out for its trapping ability and proven efficacy both in vitro and in vivo.^49–51^ In a solution with 4HNE, Phr can be expected to form thermodynamically more stable compounds. However, kinetic studies must be considered to determine which of these will combine first in the experiment.

Once the products are formed, it is important to analyze the electron transfer properties. To this end, Figure 4 shows the FEDAM of all of the compounds in water and benzene, including the adducts. In water, the adducts (black rhombuses) are close to the corresponding nitrogenous bases. In these systems, the electron acceptor capacity of 4HNE apparently has no significant influence on the electron transfer properties of the adducts. With the scavengers, adducts are close to HDC and Phr, and they are worse electron acceptors than 4HNE. For HDC in water, the compound with 4HNE is a good electron acceptor. In benzene, the results are similar, with 4HNE-guanine being the best electron donor and the worse electron acceptor. As can be seen in Figure 4, 4HNE-guanine in benzene exhibits a small negative A value. This value could become a small positive value with another base, but the conclusion would be the same; that is, 4HNE-guanine is not a good electron acceptor.

Upon analyzing the results of Figure 4, it is important to note that adducts formed with the scavenger are worse electron acceptors than 4HNE. This implies that the capacity of 4HNE to oxidize nitrogenous bases decreases when the adducts are formed. The scavenging capacity of Phr and HDC reduces the toxicity of 4HNE for two reasons: they trap 4HNE, preventing it from reacting with biomolecules, and the adducts formed are not as effective oxidants as 4HNE, resulting in less damage to biomolecules.

## Conclusions

4HNE is the main degradation product of Omega-6. Among the systems analyzed in this investigation, it is the best electron acceptor in water and benzene. It could accept electrons from nitrogenous bases, which would then be oxidized. This could be related to covalent modifications and could be linked to chronic diseases. The main degradation product of Omega-6 could be toxic due to its capacity to oxidize biomolecules.

Phr and HDC, the two scavengers of 4HNE investigated, have different electron transfer properties. HDC is a better electron donor than Phr, and both exhibit similar electron acceptor capacity. Both are good electron acceptors and good electron donors. Both scavengers could donate electrons to 4HNE. Phr and HDC have a lower capacity to oxidize nitrogenous bases than 4HNE, as they are worse electron acceptors. 4HNE exhibits potential toxicity related to its ability to oxidize biomolecules, while the scavengers (HDC and Phr) have a lower capacity to do so.

The formation of stable compounds indicates the ability of HDC and Phr to scavenge 4HNE. Phr will be a more effective scavenger than HDC since it forms stronger bonds with 4HNE. This is consistent with previous scientific evidence. All nitrogenous bases form stable compounds with 4HNE. The most stable compound is 4HNE-guanine. The stabilization energy of 4HNE-HDC in benzene is less than that of 4HNE-guanine. Therefore, in the competition with the nitrogenous bases, HDC will be less effective. These conclusions are based on thermodynamics. In future investigations, it will be important to include kinetic effects.

All adducts formed with the scavenger are worse electron acceptors than 4HNE. This implies that the capacity of 4HNE to oxidize nitrogenous bases decreases with the formation of the adducts. The scavenging capacity of Phr and HDC reduces the toxicity of 4HNE because they trap the 4HNE and the adducts formed are not as effective oxidants as those of 4HNE.

The main idea of this research is related to the crucial importance of the analysis of the degradation products that are generated. Omega-6 is beneficial for health and prevents diseases, but its main degradation product is toxic. There are substances that can bind to this toxic product, but the potential toxicity of the adducts that are formed must be investigated to analyze the potential toxicity.
